# Whether regional lymph nodes evaluation should be equally required for both right and left colon cancer

**DOI:** 10.18632/oncotarget.11007

**Published:** 2016-08-02

**Authors:** Xu Guan, Wei Chen, Zheng Liu, Zheng Jiang, Hanqing Hu, Zhixun Zhao, Song Wang, Yinggang Chen, Guiyu Wang, Xishan Wang

**Affiliations:** ^1^ Department of Colorectal Surgery, The Second Affiliated Hospital of Harbin Medical University, Harbin, China; ^2^ Follow up center, The Second Affiliated Hospital of Harbin Medical University, Harbin, China; ^3^ Department of Colorectal Surgery, Cancer Institute & Hospital, Chinese Academy of Medical Sciences, Peking Union Medical College, Beijing, China

**Keywords:** colon cancer, surgery, colectomy, lymph node, survival

## Abstract

Despite the adequacy of nodal evaluation was gradually improved for colon cancer, the disparity in nodal examination for right colon cancer (RCC) and left colon cancer (LCC) still begs the question of whether 12 nodes is an appropriate threshold for both RCC and LCC. From Surveillance, Epidemiology, and End-Results (SEER) database, we identified 53897 RCC patients and 11822 LCC patients. Compared with LCC patients, RCC patients examined more lymph nodes (18.7 vs 16.3), and more likely to examine ≥12 nodes (P<0.001), whereas RCC patients showed lower rates of node positivity (P<0.001). To balance the nodal disparity between RCC and LCC, we revised the 12-node measure based on different tumor locations. With the X-tile, we determined 15 as the optimal node number for RCC and 11 for LCC. To validate the availability of this revised nodal evaluation, the 5-year cancer specific survival (CSS) was calculated according to the optimal node number in RCC and LCC patients, Cox's regression model were used to further assess the prognostic value of this revised nodal evaluation. The results showed that 5-year CSSs were significantly improved for RCC patients with ≥15 lymph nodes, and also for LCC patients with ≥11 lymph nodes (P<0.001). This revised nodal evaluation could also improve the rate of nodal positivity and long-term survival in both RCC and LCC patients compared with 12-node measure. Therefore, the lymph node examination should be discriminately evaluated for RCC and LCC, instead of using 12-node measure to colon cancer as a whole.

## INTRODUCTION

The presence of lymph node metastasis contributes to adverse prognostic implications for colon cancer patients. Accumulating evidences have demonstrated that increasing number of lymph nodes examined for colon cancer contributed to the improvement of disease-free and overall survival [1–5]. The mechanisms underlying the potential association between lymph node count and survival outcomes remain unclear. Several potential factors may contribute to this influence on prognosis, such as accurate tumor staging, more effective surgical intervention, and superior quality of pathology service [6]. In addition, some studies have demonstrated that a greater host immune response [7], or other underlying molecular/biological characteristics of tumor, may play a role among patients with a larger lymph node count [6].

Studies have attempted to explore the optimal minimum number of lymph nodes that associated with survival outcomes, whereas individual studies varied widely in their recommendations for the number of evaluated nodes necessary to accurately determine nodal status. [4, 8–10]. Based on these studies, The American Society of Clinical Oncology (ASCO) and the National Comprehensive Cancer Network (NCCN) guideline now advocate that 12 regional lymph nodes should be the necessary minimum number for quality evaluation of colon cancer resection. However, colon cancer is one heterogeneous disease, showing variety of epidemiological, clinicopathological and molecular characteristics altered between right colon cancer (RCC) and left colon cancer (LCC) [11, 12]. Expected that, the disparity in regional lymph node examination have also been found between right colectomy and left colectomy [13, 14]. Accordingly, it may be controversial that the 12-node measure was equally applied in both RCC and LCC without consideration of different tumor locations.

Therefore, we presumed that the optimal minimum number of lymph node should be determined for RCC and LCC separately. In this work, we firstly assess the difference in regional lymph nodes evaluation between RCC and LCC. Secondly, we explored the optimal minimum number of lymph node examined according to survival benefit in RCC and LCC separately. Thirdly, we evaluated the prognostic value of the optimal number of nodes on RCC and LCC. Finally, we compared the rate of node positivity and long-term survival between the standard 12-node measure and the revised node measure in both RCC and LCC.

## RESULTS

### Patient characteristics

From the SEER database, we totally identified 65719 colon cancer patients including 53897 RCC patients and 11822 LCC patients. RCC were more common seen in female patients (54.8%), whereas LCC were more frequently in male patients (53.9%). Patients aged more than 60 were accounted for 79.6% in RCC, and 66.1% in LCC. Most of the patients were white in both RCC (81.5%) and LCC patients (75.2%). The proportion of Stage III in LCC was 39.1%, which was obviously larger than RCC patients (35.1%). RCC patients demonstrated poor differentiation more frequently than LCC patients. Additionally, only 8.2% of LCC patients were diagnosed with mucous/signet-ring cell tumor, compared with 13.7% in RCC patients. All characteristics had significant differences between RCC and LCC, with P<0.001. The detailed characteristic information was shown in Table 1.

**Table 1:** Comparisons of clinical characteristics among RCC and LCC patients

Characteristic	Right colon cancer		Left colon cancer		P
**Gender**					
**Male**	24357	45.2%	6370	53.9%	<0.001
**Female**	29540	54.8%	5452	46.1%	
**Age at diagnosis, year**					<0.001
**20-59**	11013	20.4%	4001	33.9%	
**≥60**	42884	79.6%	7821	66.1%	
**Race**					
**White**	43920	81.5%	8892	75.2%	<0.001
**Black**	6353	11.8%	1801	15.2%	
**Others**	3624	6.7%	1129	9.6%	
**AJCC stage**					
**Stage I**	13593	25.2%	2707	22.9%	<0.001
**Stage II**	21369	39.7%	4489	38.0%	
**Stage III**	18935	35.1%	4626	39.1%	
**AJCC T stage**					
**T1**	6358	11.8%	1659	14.0%	<0.001
**T2**	9334	17.3%	1604	13.6%	
**T3**	32282	59.9%	7221	61.1%	
**T4**	5923	11.0%	1338	11.3%	
**AJCC N stage**					
**N0**	34962	64.9%	7196	60.9%	<0.001
**N1/2**	18935	35.1%	4626	39.1%	
**Tumor grade**					
**1/2**	40173	74.5%	9733	82.3%	<0.001
**3/4**	12182	22.6%	1681	14.2%	
**Unknown**	1542	2.9%	408	3.5%	
**Histology**					
**Adenocarcinoma**	46053	85.5%	10800	91.4%	<0.001
**Mucous/signet-ring cell**	7409	13.7%	974	8.2%	
**Others**	435	0.8%	48	0.4%	

### Lymph node comparisons between RCC and LCC

**Figure 1:**
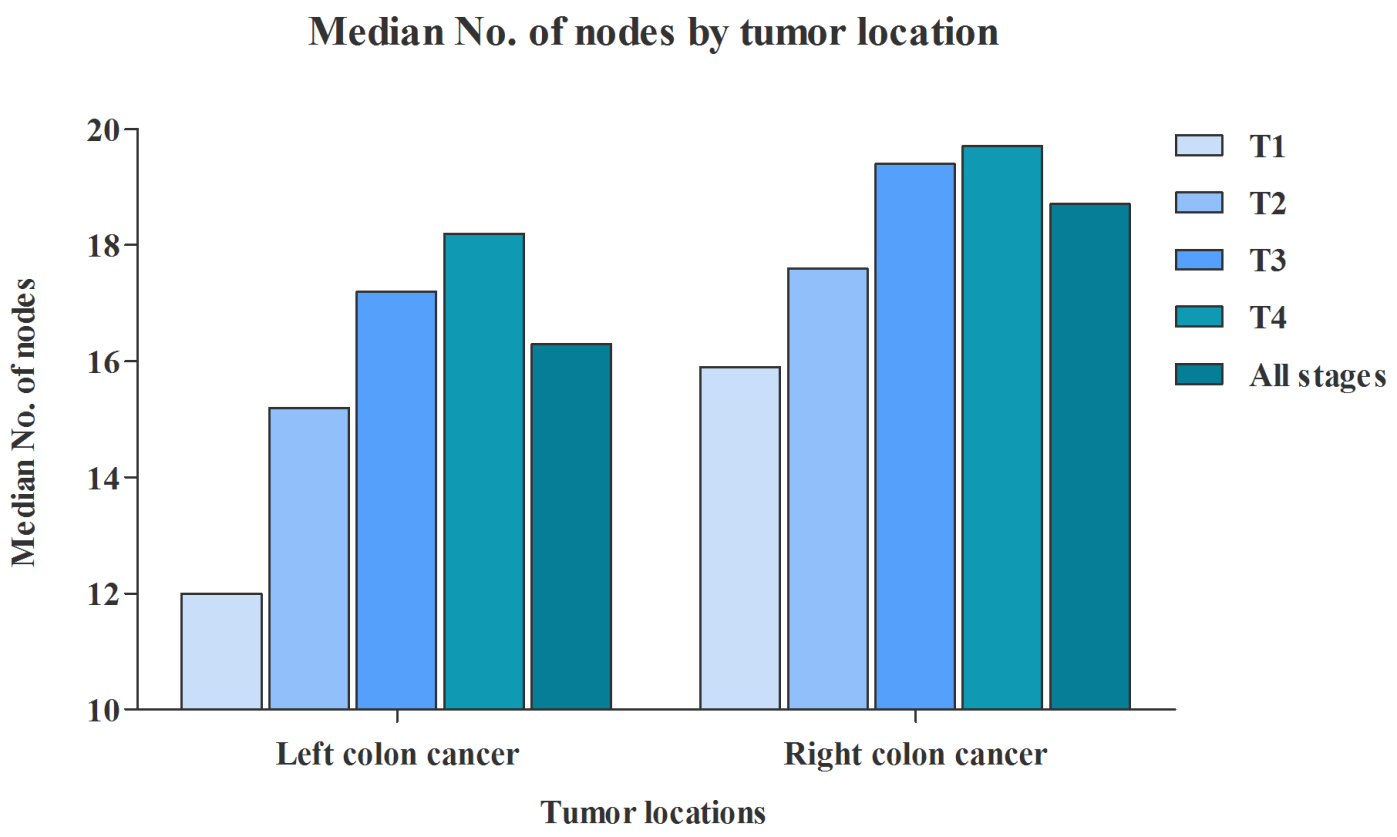
Comparison of median No. of nodes between RCC and LCC

**Figure 2:**
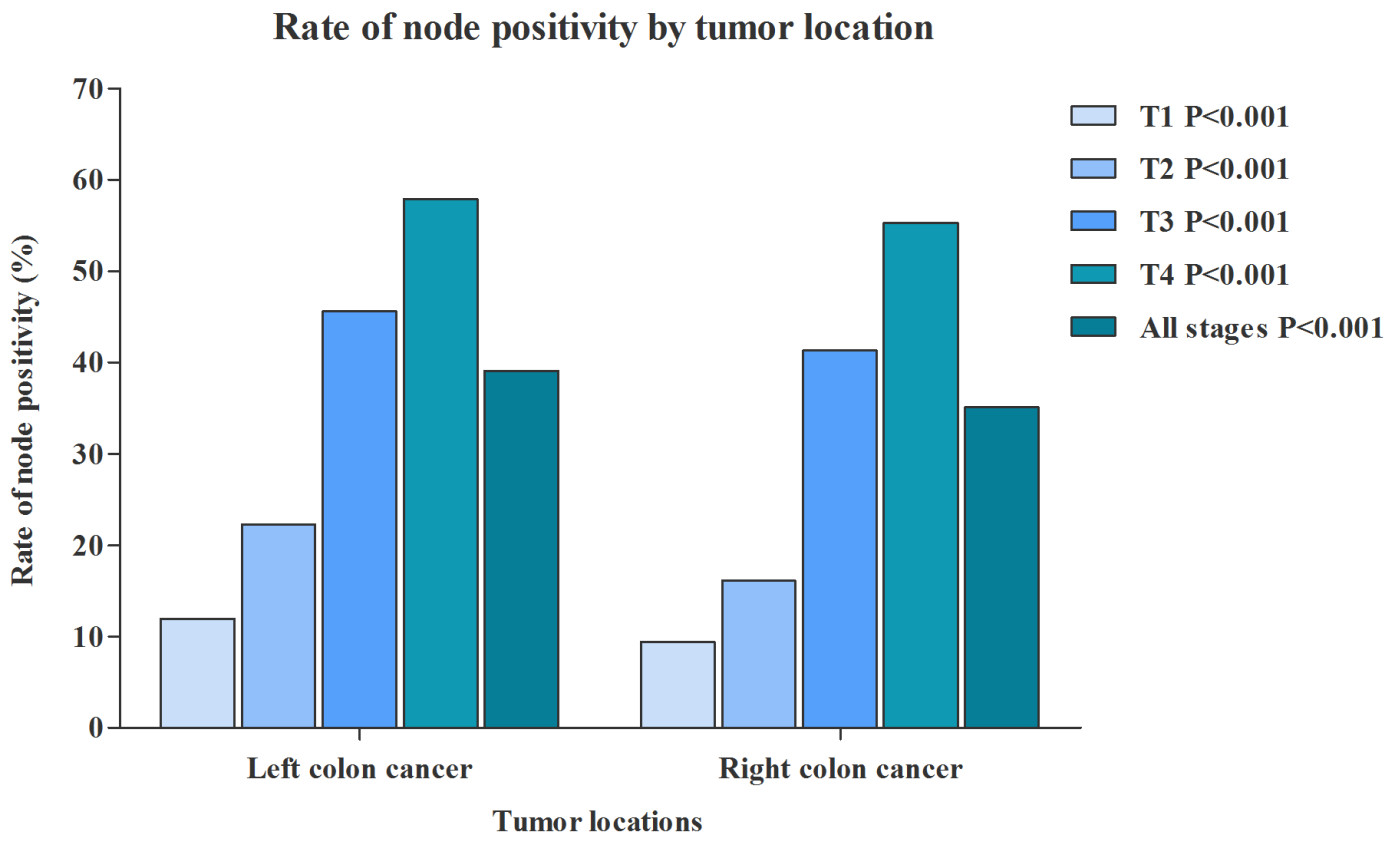
Comparison of rate of node positivity between RCC and LCC

**Figure 3:**
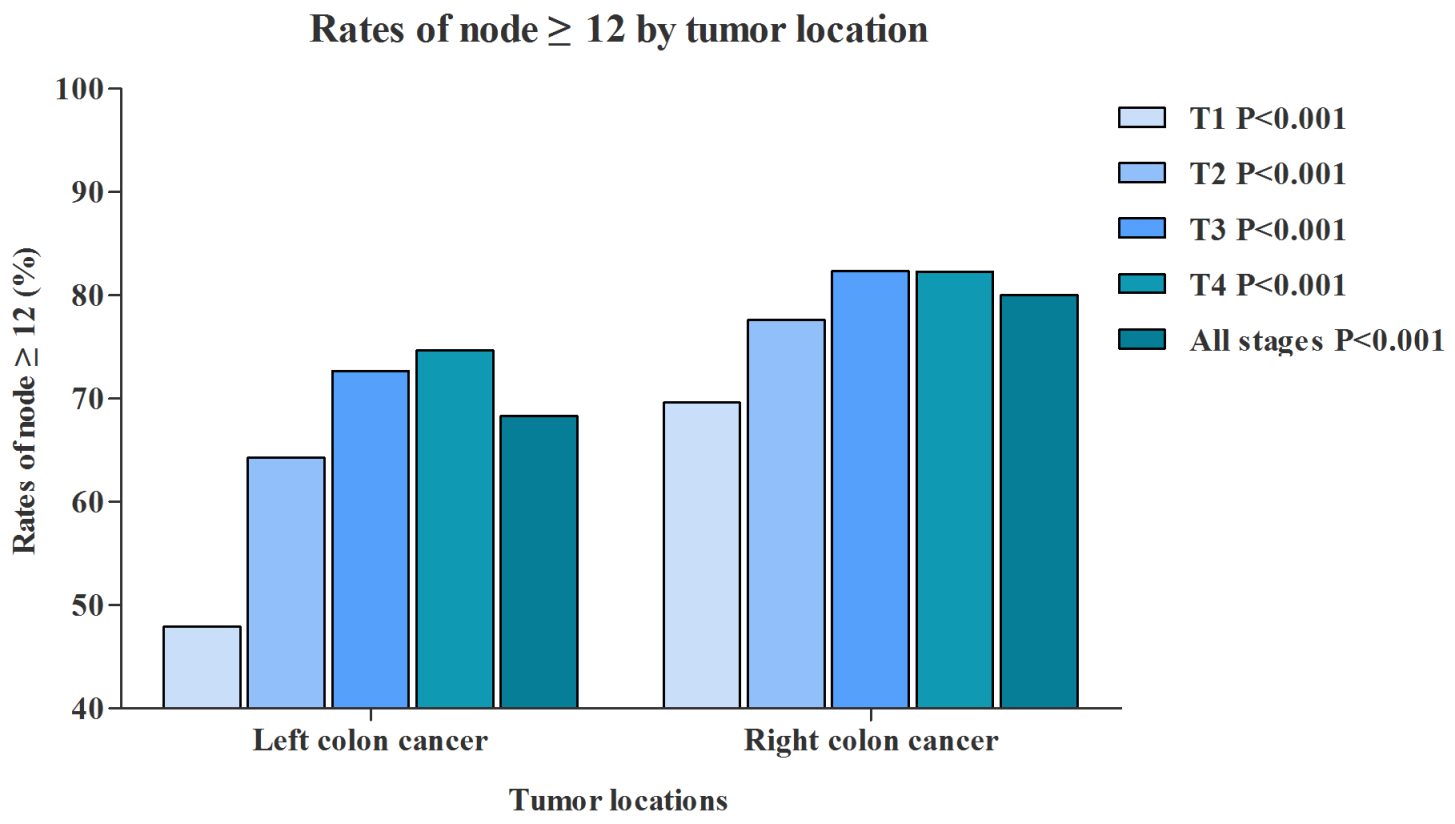
Comparison of rate of nodes ≥12 between RCC and LCC

RCC patients had a median of 18.7 lymph nodes examined, and LCC patients had 16.3 lymph nodes examined. In addition, we further evaluated the median of lymph nodes examined in different T stage separately (Figure 1). With the T stage increased, the median of lymph nodes examined were increased from 15.9 to 19.1 in RCC patients, from 12.0 to 18.2 in LCC patients. Although more lymph nodes examined in RCC, the proportion of RCC patients with node positivity was 35.1%, which was significantly lower than LCC patients (39.1%) (Figure 2). The association between T stage and rate of node positivity was also presented with positive correlation. Furthermore, compared with LCC patients, RCC patients were more frequently examined with more than 12 lymph nodes (Figure 3). The rates of more than 12 lymph nodes also increased with T stage in both RCC and LCC patients.

Compared with LCC, RCC examined more lymph nodes, and was much easier to meet the requirement of 12 nodes examined. However, RCC patients were presented with lower rates of node positivity, which could contribute to the understaging, in which inadequate evaluation might incorrectly identify patients with node-positive as node-negative, thus failing to select appropriate treatment. To balance the disparity of nodal evaluation between RCC and LCC, we suggested to either/both increase the number of lymph nodes examined for RCC or/and decrease the number of lymph nodes examined for LCC.

### Identification of the optimal cutoff points of lymph nodes in RCC and LCC

**Figure 4:**
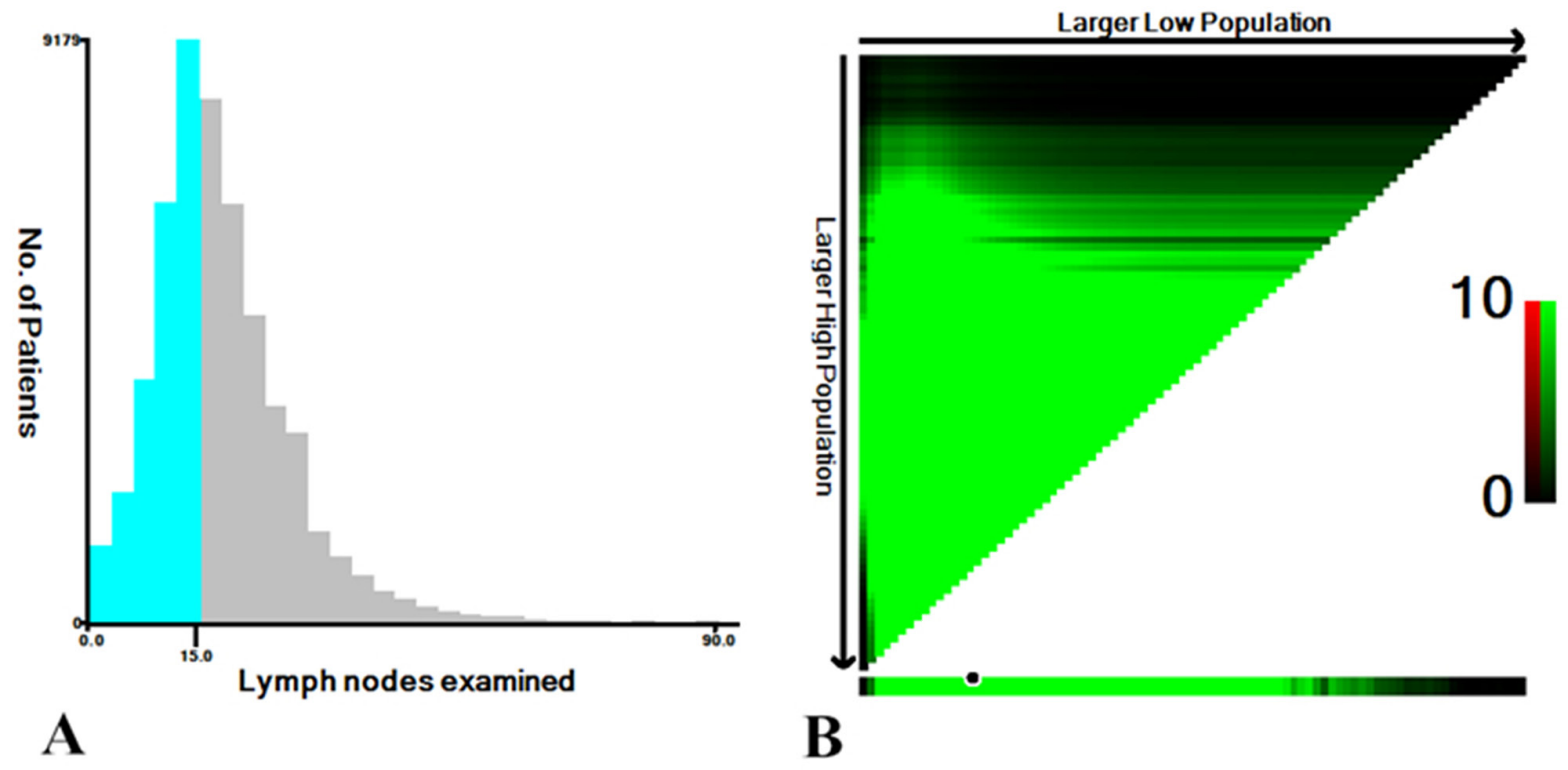
**A.** The distribution of number of RCC patients according to lymph nodes examined. No. of lymph nodes ranged from 0 to 90. **B.** X-tile plots for No. of lymph nodes constructed by RCC patients. The plots show the χ^2^ log-rank values produced, dividing them into 2 groups by the cutoff point 15. The brightest pixel represents the maximum χ^2^ log-rank value.

**Figure 5:**
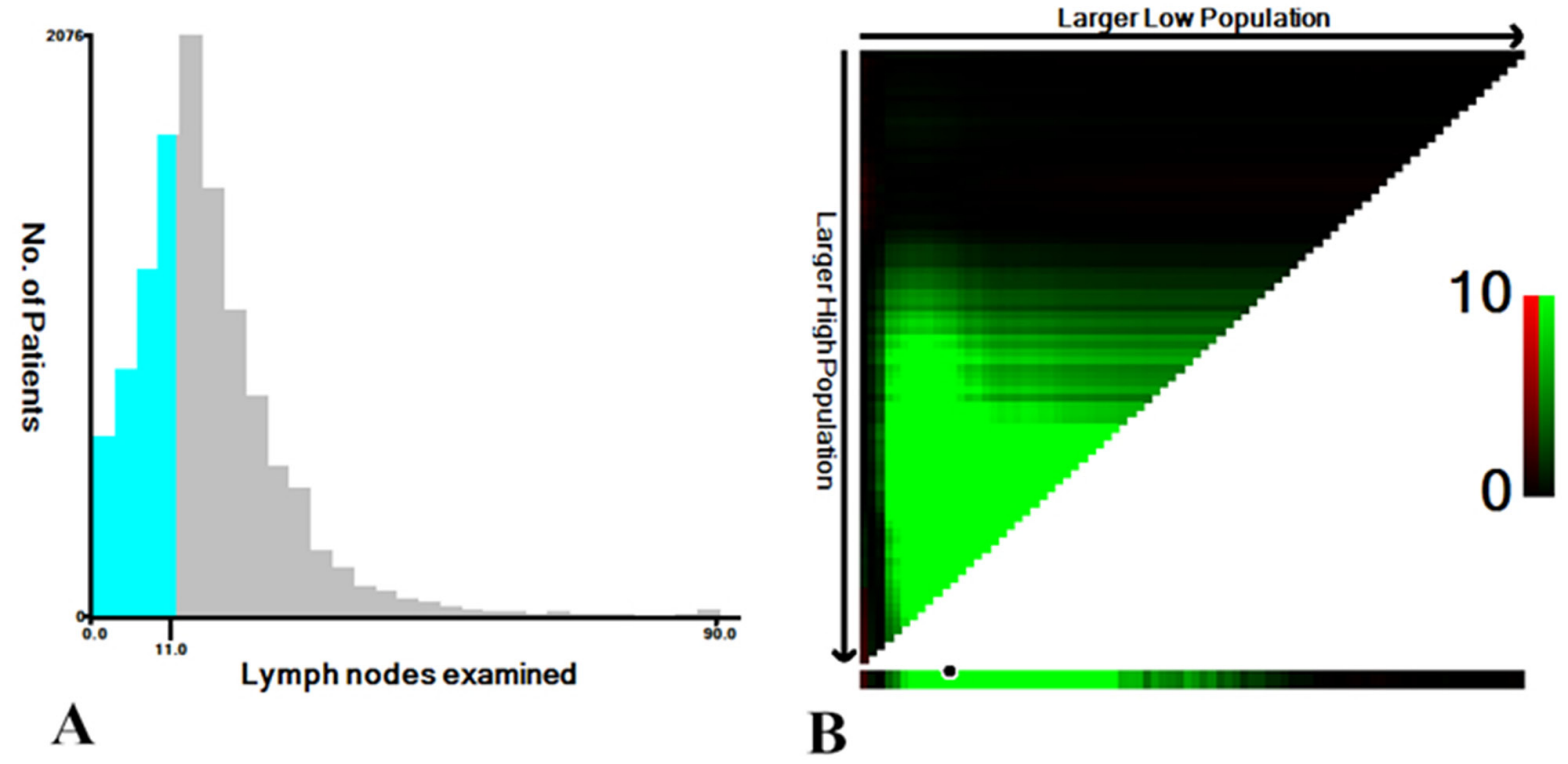
**A.** The distribution of number of LCC patients according to lymph nodes examined. No. of lymph nodes ranged from 0 to 90. **B.** X-tile plots for No. of lymph nodes constructed by LCC patients. The plots show the χ^2^ log-rank values produced, dividing them into 2 groups by the cutoff point 11. The brightest pixel represents the maximum χ^2^ log-rank value.

X-tile was used to determine the optimal cutoff point for prediction of CSS according to the number of lymph nodes examined. X-tile analysis indicated that the maximum χ^2^ log-rank value of 379.1 was produced determining 15 as cutoff value to divide RCC patients with the strongest discriminatory capacity (P<0.001) (Figure 4). With the same method, we explored the optimal cutoff value 11 for LCC patients, corresponding to the maximum of χ^2^ log-rank value of 31.9 (P<0.001) (Figure 5). Therefore, the two cutoff values were separately used as prognostic factors for further analysis in RCC and LCC patients.

Then, we performed the comparison of rate of node ≥new cutoff values (11 for LCC/15 for RCC) between RCC and LCC (Supplementary Figure S1). We found that the rates of node ≥15 for RCC patients were much similar to the rates of node ≥11 for LCC patients compared with the standard 12-node measure. This result implied that the nodal disparity between RCC and LCC was well balanced with the application of the revised measure.

### Association between the optimal lymph nodes and node positivity

For RCC patients, multivariate analyses demonstrated that RCC patients with ≥15 lymph nodes were significantly more likely to have node-positive disease (odds ratio [OR] for ≥15 nodes vs <15 nodes: 1.392; 95% CI: 1.349-1.437) (Table 2). For LCC patients, the patients with ≥11 lymph nodes evaluation were more likely to have node-positive disease compared with those with <11 nodes evaluated (OR for > nodes 11 vs <11 nodes, 1.355; 95% CI, 1.245-1.474) (Table 3). These results indicated that revised nodal evaluation could improve the rate of nodal positivity in both RCC and LCC patients. In addition, we also found there was no obvious difference of node positivity rate between patients with <12 nodes and patients with ≥12 nodes for both LCC and RCC (Supplementary Table S1 and Supplementary Table S2).

**Table 2:** Relative odds of node positivity among RCC patients

Characteristic		OR [95% CI]	P
**Nodes examined**	<15	1	<0.001
	≥15	1.392 [1.349-1.437]	
**Gender**	Female	1	0.633
	Male	0.991 [0.954-1.029]	
**Age**	20-59	1	<0.001
	≥60	0.747 [0.714-0.781]	
**Race**	White	1	<0.001
	Black	1.186 [1.119-1.257]	
	Others	1.129 [1.048-1.215]	
**AJCC T Stage**	T1	1	<0.001
	T2	1.378 [1.255-1.513]	
	T3	4.458 [4.118-4.825]	
	T4	8.439 [7.691-9.260]	
**Histological type**	Adenocarcinoma	1	<0.001
	Mucous/signet-ring cell	1.073 [1.017-1.131]	
	Others	0.846 [0.690-1.037]	
**Grade**	Grade I/II	1	<0.001
	Grade III/IV	1.975 [1.891-2.064]	

**Table 3:** Relative odds of node positivity among LCC patients

Characteristic		OR [95% CI]	P
**Nodes examined**	<11	1	<0.001
	≥11	1.355 [1.245-1.474]	
**Gender**	Female	1	0.039
	Male	0.925 [0.858-0.996]	
**Age**	20-59	1	<0.001
	≥60	0.847 [0.783-0.916]	
**Race**	White	1	<0.001
	Black	1.155 [1.041-1.281]	
	Others	1.297 [1.143-1.471]	
**AJCC T Stage**	T1	1	<0.001
	T2	1.331 [1.080-1.641]	
	T3	1.653 [1.428-1.913]	
	T4	1.627 [1.294-2.047]	
**Histological type**	Adenocarcinoma	1	<0.001
	Mucous/signet-ring cell	1.279 [1.118-1.463]	
	Others	0.512 [0.272-0.963]	
**Grade**	Grade I/II	1	<0.001
	Grade III/IV	1.497 [1.345-1.666]	

### Survival benefit of the revised nodal evaluation in RCC and LCC patients

**Figure 6:**
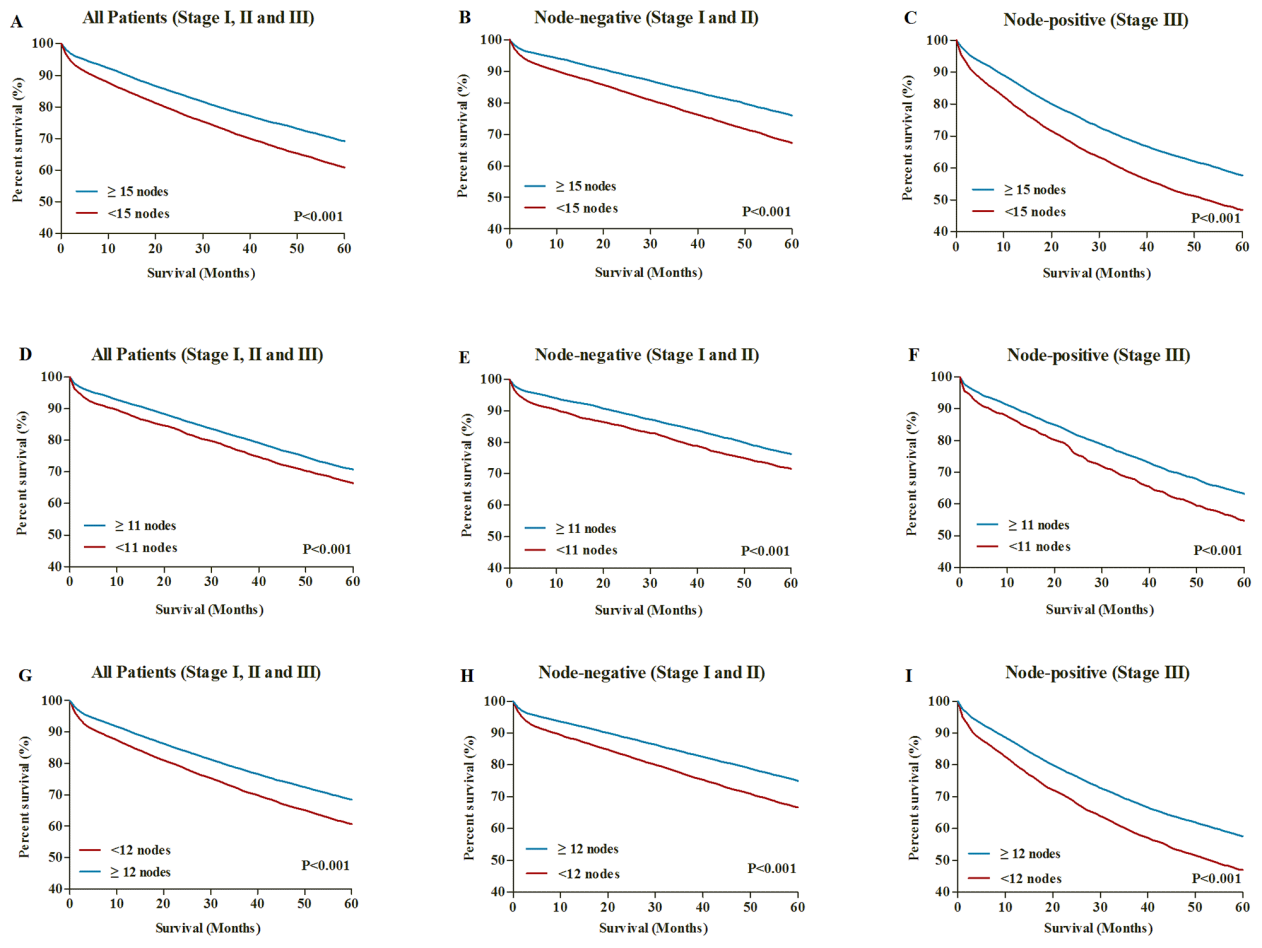
**A.** 5-year CSSs in all RCC patients with lymph node ≥15 and <15. **B.** 5-year CSSs in node-negative RCC patients with lymph node ≥15 and <15. **C.** 5-year CSSs in node-positive RCC patients with lymph node ≥15 and <15. **D.** 5-year CSSs in all LCC patients with lymph node ≥11 and <11. **E.** 5-year CSSs in node-negative LCC patients with lymph node ≥11 and <11. **F.** 5-year CSSs in node-positive LCC patients with lymph node ≥11 and <11. **G.** 5-year CSSs in all patients with lymph node ≥12 and <12. **H.** 5-year CSSs in all node-negative patients with lymph node ≥12 and <12. **I.** 5-year CSSs in all node-positive patients with lymph node ≥12 and <12.

The 5-year CSS was 69.1% for RCC patients with lymph nodes ≥15 and 61.3% for those with lymph nodes <15 (P<0.001) (Figure 6A). The 5-year CSS was 70.7% for LCC patients with lymph nodes ≥11 and 66.4% for those with lymph nodes <11 (P<0.001) (Figure 6D). To avoid the potential influence of lymph status, we stratified patients to examine the relationship between nodal evaluation and survival among those with either node-negative (AJCC stage I and II) or node-positive (AJCC stage III) disease. The results indicated that RCC patients in both node-negative and node-positive could had survival benefit from ≥15 nodes examined, which were significantly better than patients with <15 nodes examined (Figure 6B and 6C). Similarly, we also found that LCC patients with ≥ 11 nodes examined could obtain survival benefit, regardless of nodal status (Figure 6E and 6F). Furthermore, we performed the comparisons of 5-year CSSs between patients with ≥12 nodes and patients with <12 nodes (Figure 6G, 6H and 6I). Although there has significant differences of 5-year CSSs between patients with ≥12 nodes and patients with <12 nodes, this 12-node measure took LCC and RCC as a whole and might not distinguish the prognostic differences between LCC and RCC.

### Identifying the survival risk factors in RCC and LCC patients

With univariate and multivariate regression analyses, we further identified the patient- and tumor-associated risk factors that associated with long-term survival outcomes in RCC and LCC patients separately. The results showed that examining <15 nodes was identified as independent adverse prognostic factors in RCC patients (Table 4), examining <11 node was also considered as risk factor for LCC patients (Table 5). In addition, characteristics including age≥60, stage II/III, T2/3/4 stage, N1/2 stage, grade III/IV and mucous/signet-ring cell cancer were all identified independent adverse prognostic factors in both RCC and LCC patients. Here, we also performed the Cox's regression analysis again based on the cutoff of 12 nodes. The results showed that examining <12 nodes were considered as adverse prognostic factor for long-term survival in both LCC and RCC. (Supplementary Table S3 and Supplementary Table S4).

**Table 4:** Univariate and multivariate analyses for RCC patients

Characteristic		Univariate analysis		Multivariate analysis	
		HR [95% CI]	P	HR [95% CI]	P
**Nodes examined**	<15	1	<0.001	1	<0.001
	≥15	0.733 [0.710-0.756]		0.714 [0.692-0.736]	
**Gender**	Female	1	0.975		
	Male	0.999 [0.969-1.031]			
**Age**	20-59	1	<0.001	1	<0.001
	≥60	2.435 [2.316-2.561]		2.531 [2.405-2.662]	
**Race**	White	1	<0.001	1	<0.001
	Black	0.955 [0.909-1.002]		1.084 [1.033-1.139]	
	Others	0.697 [0.649-0.749]		0.709 [0.660-0.762]	
**AJCC Stage**	Stage I	1	<0.001	1	<0.001
	Stage II	1.436 [1.373-1.503]		1.493 [1.418-1.476]	
	Stage III	2.335 [2.235-2.440]		2.435 [2.289-2.645]	
**AJCC T Stage**	T1	1	<0.001	1	<0.001
	T2	1.241 [1.156-1.333]		1.218 [1.133-1.310]	
	T3	1.842 [1.734-1.957]		1.977 [1.782-2.194]	
	T4	3.451 [3.222-3.696]		3.406 [3.055-3.797]	
**AJCC N Stage**	N0	1	<0.001	1	<0.001
	N1/2	1.848 [1.792-1.907]		1.329 [1.157-1.528]	
**Histological type**	Adenocarcinoma	1	<0.001	1	<0.001
	Mucous/signet-ring cell	1.156 [1.109-1.206]		1.053 [1.009-1.099]	
	Others	1.917 [1.592-2.309]		1.541 [1.277-1.858]	
**Grade**	Grade I/II	1	<0.001	1	<0.001
	Grade III/IV	1.496 [1.446-1.549]		1.244 [1.181-1.268]	

**Table 5:** Univariate and multivariate analyses for LCC patients

Characteristic		Univariate analysis		Multivariate analysis	
		HR [95% CI]	P	HR [95% CI]	P
**Nodes examined**	<11	1	<0.001	1	<0.001
	≥11	0.820 [0.762-0.882]		0.730 [0.678-0.787]	
**Gender**	Female	1	0.121		
	Male	1.057 [0.986-1.133]			
**Age**	20-59	1	<0.001	1	<0.001
	≥60	2.413 [2.209-2.637]		2.556 [2.338-2.796]	
**Race**	White	1	<0.001	1	<0.001
	Black	1.124 [1.023-1.235]		1.224 [1.113-1.346]	
	Others	0.738 [0.644-0.846]		0.741 [0.646-0.850]	
**AJCC Stage**	Stage I	1	<0.001	1	<0.001
	Stage II	1.644 [1.477-1.829]		1.771 [1.615-1.967]	
	Stage III	2.246 [2.025-2.491]		2.014 [1.748-2.375]	
**AJCC T Stage**	T1	1	<0.001	1	<0.001
	T2	1.430 [1.212-1.686]		1.468 [1.238-1.740]	
	T3	2.113 [1.851-2.412]		2.471 [1.955-3.124]	
	T4	4.041 [3.477-4.697]		4.575 [3.582-5.843]	
**AJCC N Stage**	N0	1	<0.001	1	0.158
	N1/2	1.579 [1.473-1.693]		1.185 [0.936-1.499]	
**Histological type**	Adenocarcinoma	1	<0.001	1	0.314
	Mucous/signet-ring cell	1.292 [1.152-1.448]		1.094 [0.975-1.228]	
	Others	1.119 [0.674-1.858]		1.007 [0.604-1.678]	
**Grade**	Grade I/II	1	<0.001	1	<0.001
	Grade III/IV	1.541 [1.410-1.685]		1.331 [1.216-1.458]	

### The comparisons of rate of node positivity between 12-node measure and the revised measure for LCC and RCC

For LCC patients, the rates of node positivity were 42.9% and 42.7% for 12-node measure and 11-node measure (Supplementary Figure S2A). There also have no differences of rate of node positivity in T1, T2, T3 and T4 stages. Therefore, despite one node was decreased for 11-node measure compared with 12-node measure, no difference of rate of node positivity was detected between two measures. For RCC patients, the rate of node positivity was obviously increased from 35.5% for 12-node measure to 38.3% for 15-node measure (Supplementary Figure S2B). The increased rates of node positivity could be detected in T1, T2, T3 and T4 stages. These results suggested that the 15-node measure could improve the rate of node positivity compared with 12-node measure for RCC patients.

### The comparisons of long-term survival between 12-node measure and the revised measure for LCC and RCC

For LCC patients, the 3-year CSS, 5-year CSS and 8-year CSS were 81.2%, 70.9% and 61.7% for patients with ≥12 nodes, the 3-year CSS, 5-year CSS and 8-year CSS were 81.1%, 70.7% and 61.2% for patients with ≥11 nodes (Supplementary Figure S3A). We found that there were no differences of long-term survival between two measures for LCC. For RCC patients, the 3-year CSS, 5-year CSS and 8-year CSS were 77.9%, 67.9% and 56.8% for patients with ≥12 nodes, the 3-year CSS, 5-year CSS and 8-year CSS were increased to 79.0%, 69.1% and 58.2% for patients with ≥15 nodes (Supplementary Figure S3B). Therefore, compared with 12-node measure, the 15-node measure could improve the long-term survival for RCC patients.

## DISCUSSION

Although the number of lymph nodes evaluated for colon cancer has markedly increased in the past 2 decades [15], differences in nodal evaluation between RCC and LCC were continue to exist over time. Previous studies have indicated that the median number of nodes examined was significantly lower for LCC compared with RCC. Karl et al. identified 142009 N0M0 colon cancer patients from National Cancer Data Base (1998-2004), and the median number of nodes examined was 12 for RCC and 8 for LCC [13]. In this study, we also found that the median number of nodes examined was significantly different between RCC and LCC (18.7 vs. 16.3). Furthermore, previous studies reported that patients with colon cancers were only 55%-59% likely to examine lymph node ≥12 [13, 14]. Here, we found that up to 80% of RCC patients were likely to examined nodes ≥12, which was obviously higher than LCC patients (68.3%). Our study only included the patients who underwent hemicolectomy and excluded the patients who underwent segmental resection, which resulted in more lymph nodes examined here than other studies.

The potential reasons for more lymph nodes examined on RCC than the LCC were multifactorial. Firstly, surgical quality measure might play an important role in the variability of node evaluation between RCC and LCC. The extent of the colectomy by surgeons directly affects the number of lymph nodes examined, It was common known that the resection margins of right hemicolectomy was standard and uniform for RCC, including terminal ileum, cecum, ascending colon, hepatic flexure and half of transverse colon. In contrast, the extent of colectomy recommended for LCC was closely based on the tumor location within the left colon, varying from formal left hemicolectomy to segmental resection. Therefore, the resection extent of RCC was larger than LCC, which might contribute to more lymph node examined on RCC compared with LCC [13]. In this study, patients who underwent the segmental resection were excluded to avoid the influence of the resection extent on lymph node examination, and the gap of lymph nodes examined between RCC and LCC was obviously decreased compared with previous studies [13, 14].

Secondly, the adequacy of assessment of lymph nodes by pathologist may affect the number of lymph nodes examined, the pathologic examination was associated with accountability at different hospitals or pathologists level [16]. This was why many techniques, such as lymph node-revealing solution, fat clearance with alcohol and xylene and the increased application of pathology template, have all been considered as improving lymph node evaluation [16–18]. However, this was not the reason for the variability in nodal examination between RCC and LCC, because it was impossible that the pathologist use different techniques with different degrees of diligence between RCC and LCC, they would be affected equally.

Thirdly, the differences of immune response and molecular features between RCC and LCC should also be considered as influential factors for the number of lymph nodes examined, right colon and left colon involved different features related to immune response, anatomical, physiological, and molecular characteristics [19]. The benefits related to more lymph nodes examined might reflect the host lymphocytic reaction to tumor, which was associated with lymph node count [4, 20]. This phenomenon was commonly observed in lymph node draining cancer. Lower immune response may contribute to smaller lymph nodes, and a lower number being identified. It was supposed that the right colon mesentery might anatomically contain more complex lymphatic system which leads to an enhanced immune response and increased lymph nodes examined for RCC, but there was no clear evidence for this issue, and the cadaveric study may be helpful to further elaborate this [13].

Adjuvant chemotherapy plays an important role in the treatment of non-distant metastatic colon cancer [21]. The nodal status was considered as a determining factor for colon cancer patients to receive adjuvant chemotherapy. Patients with lymph node-positive disease were more likely to receive adjuvant chemotherapy [22, 23]. However, due to the lack of chemotherapy information in SEER database, the potential confounding effect of the chemotherapy may not be assessed for RCC and LCC separately. To take this potential limitation into account, we stratified our results by nodal status to examine the relationship between the optimal nodal evaluation and 5-year CSS among those with either node-negative or node-positive disease. Our results indicated that the revised nodal evaluation could be used to evaluate the long-term survival in both node-negative and node-positive disease.

Although the strengths of this study including large sample size, many limitations should be explained. First of all, the SEER database lacked some tumor- and treatment-related information, such as angiolymphatic invasion, margin of resection and patient comorbidities. All these factors were closely associated with the long-term survival of CC patients. Secondly, the SEER database collected cancer data from population-based cancer registries covering approximately 30 percent of the US population, which lead to large variations of surgical procedures and pathologic techniques for detecting lymph nodes. Therefore, the effect of surgical and pathologic technique on lymph node examination couldn't be analyzed in this large population-based study. Finally, this study was retrospective and lack homogeneity, which warranted further studies in prospective randomized trials to prove the availability of this procedure in present study.

In the light of the above considerations, the number of lymph node examined of RCC is still higher than LCC, despite the fact that nodal counts for both RCC and LCC were increased with time. The lymph node examination should be discriminately evaluated between RCC and LCC, instead of applying 12 nodes measure to both RCC and LCC. It is reasonable to either/both increase the number of lymph nodes examined for RCC or/and decrease the number of lymph nodes examined for LCC. In present study, the cutoff values 15 and 11 might be more appropriate for patients with RCC and LCC.

## MATERIALS AND METHODS

### Data resources

We extracted cancer data from the Surveillance, Epidemiology, and End Results (SEER) database [24]. The SEER collected and published the cancer incidence, treatment and survival data from 17 population-based cancer registries, which covered approximately 28 percent of the US population. The SEER database is considered to be the representative of the US population as a whole. The SEER database is an openly accessed database, cancer cases and population information could be obtained from the SEER. Data collected from the SEER database do not require informed patient consent, because they were anonymized and de-identified prior to release. We have got permission to access the cancer data from the SEER database by National Cancer Institute, and the reference number was 11228-Nov2014. This study was approved by the Second Affiliated Hospital of Harbin Medical University institutional review board.

### Study population

We identified patients older than 20 years who were diagnosed their first invasive colon cancer in stage I to stage III from January 1, 2004 to December 31, 2012. Patients included in this study should undergo radical resection of the colon cancer as the first course of therapy, which were more available and accurate for the lymph node evaluation. RCC included tumors being located at cecum, ascending colon, hepatic flexure and transverse colon. LCC included tumors being located at splenic flexure, descending colon and sigmoid colon. Excluded from our study included patients who dead due to other causes, patients with an unknown number of nodes examined, patients who received preoperative radiotherapy in the consideration of the decreased number of node examined, and patients who underwent a local procedure, partial colon resection or total colectomies.

### Statistical analysis

Firstly, we evaluated differences in patient characteristics, lymph node evaluations and node positivity between RCC and LCC using the χ^2^ test. We compared the differences of lymph nodes evaluations between RCC and LCC in three ways: 1) Median number of lymph node examined. 2) Rate of node positivity, with at least 1 positive lymph node examined as lymph node-positive disease. 3) Rate of 12 or more lymph nodes examined, 12 or more than lymph nodes was considered as adequate nodal evaluation in current guidelines.

Furthermore, we revised the current 12 nodes measure, and separately identified the optimal number of lymph node for RCC and LCC with X-tile, using the minimum P values from log-rank χ^2^ statistics. Logistic regression model was performed to estimate the association between the optimal lymph node and relative odds of node positivity among RCC and LCC patients.

Finally, to validate the survival benefit of the revised nodal evaluation in RCC and LCC, the 5-year cancer specific survival (CSS) was calculated with Kaplan-Meier method according to the optimal number of lymph node identified in RCC and LCC patients, and log-rank tests were used to compare the differences of CSS curves. Univariate and multivariate Cox's regression model were analyzed to examine hazard rate (HR) and exact 95% confidence intervals (CIs), which were further used to compare the prognosis benefit of this revised nodal evaluation. P<0.05 (two sides) was considered to be statistical significance. The statistical analyses were performed by using SPSS statistical software, version 20 (IBM Corp, Armonk, NY, USA).

## SUPPLEMENTARY FIGURES AND TABLES


