# Supplementary material for: Whether regional lymph nodes evaluation should be equally required for both right and left colon cancer
Source: Oncotarget. 2016 Aug 2;7(37):59945–56. doi: 10.18632/oncotarget.11007 (PMC5312360; doi:10.18632/oncotarget.11007)
Supplement: Supplementary file 1 [file oncotarget-07-59945-s001.pdf]

Whether regional lymph nodes evaluation should be equally required for both right and left colon cancer

SUPPLEMENTARY FIGURES AND TABLES

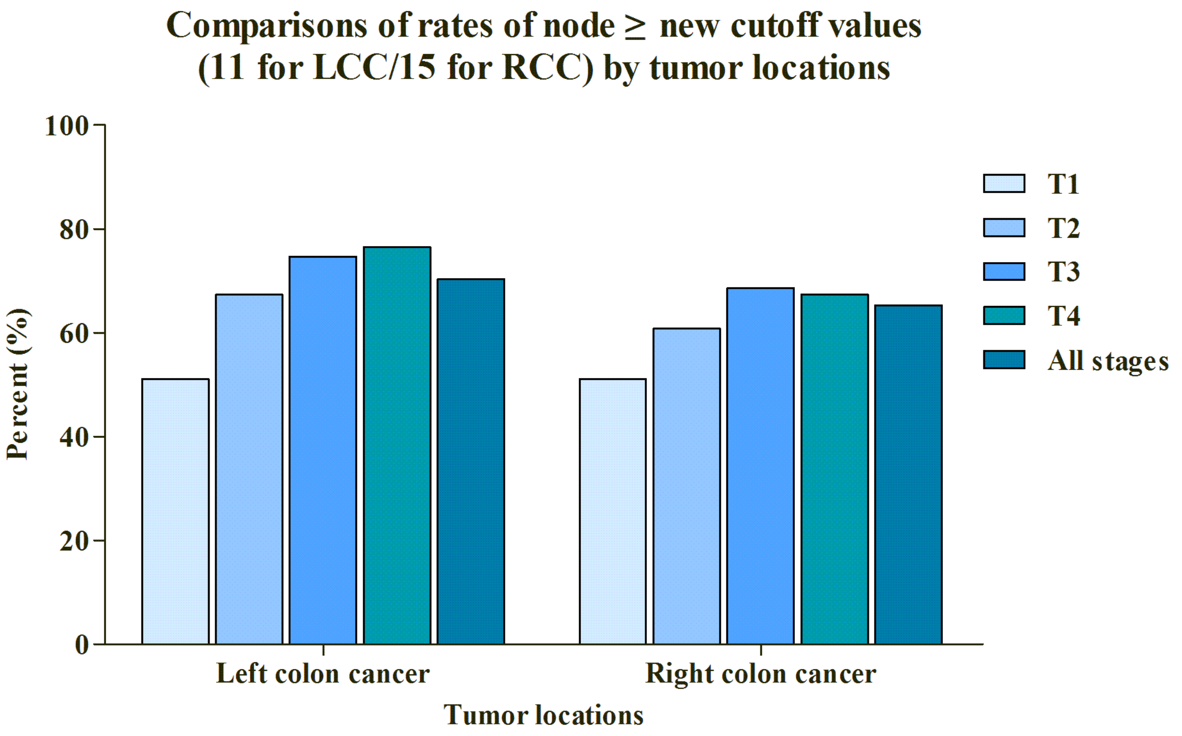

Supplementary Figure S1: Comparison of rate of node ≥ new cutoff values between RCC and LCC.

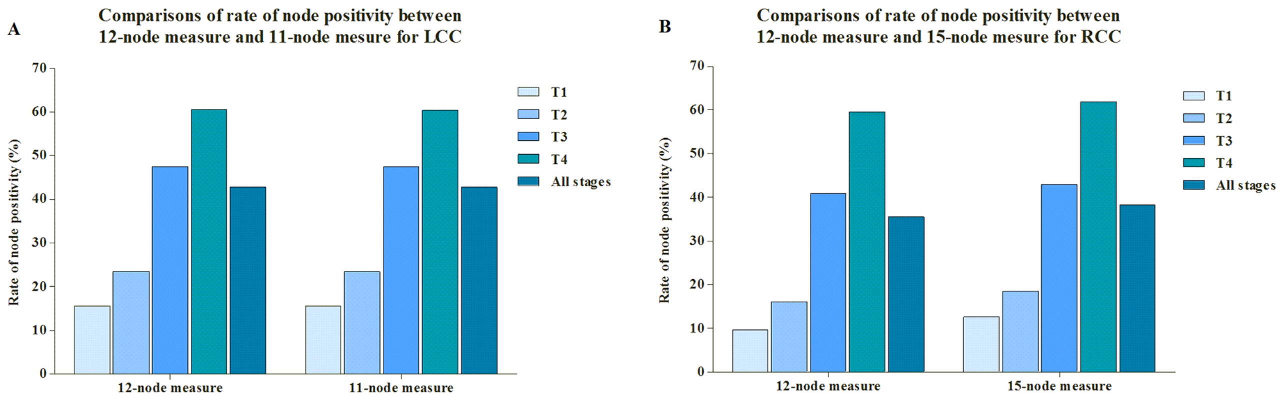

Supplementary Figure S2: A. The comparisons of rate of node positivity between 12-node measure and 11-node measure for LCC. **B.** The comparisons of rate of node positivity between 12-node measure and 15-node measure for RCC.

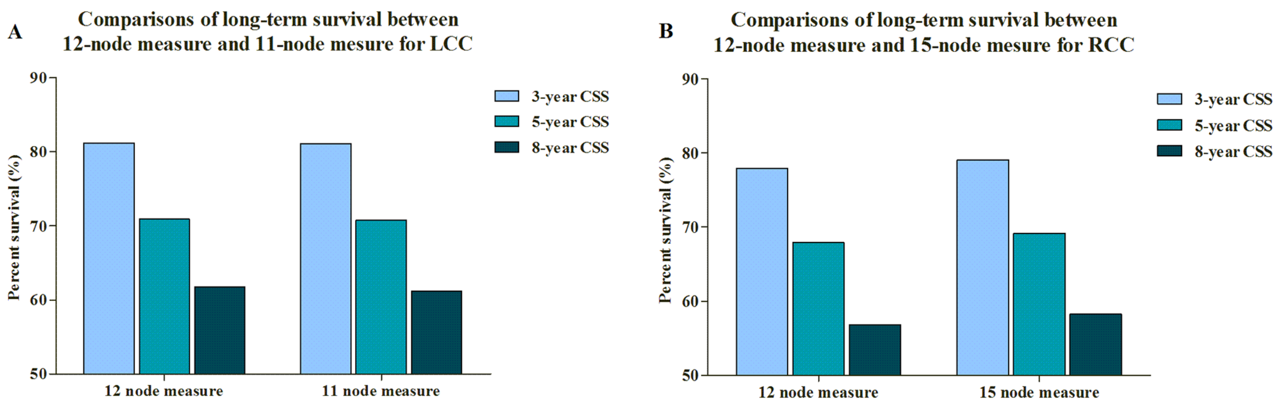

Supplementary Figure S3: A. The comparisons of long-term survivals between 12-node measure and 11-node measure for LCC. **B.** The comparisons of long-term survivals between 12-node measure and 15-node measure for RCC.

Supplementary Table S1: Relative odds of node positivity among RCC patients: multivariate logistic regression

Characteristic		OR [95% CI]	P
**Node examined**	<12	1	0.376
	≥12	0.979 [0.933-1.027]	
**Gender**	Female	1	0.640
	Male	0.991 [0.954-1.029]	
**Age**	20-59	1	<0.001
	≥60	0.735 [0.702-0.770]	
**Race**	White	1	<0.001
	Black	1.186 [1.119-1.258]	
	Others	1.128 [1.048-1.215]	
**AJCC T Stage**	T1	1	<0.001
	T2	1.415 [1.288-1.554]	
	T3	4.581 [4.229-4.963]	
	T4	8.721 [7.943-9.575]	
**Histological type**	Adenocarcinoma	1	0.008
	Mucous/signet-ring cell	1.070 [1.014-1.128]	
	Others	0.831 [0.679-1.019]	
**Grade**	Grade I/II	1	<0.001
	Grade III/IV	1.972 [1.887-2.060]	

Supplementary Table S2: Relative odds of node positivity among LCC patients: multivariate logistic regression

Characteristic		OR [95% CI]	P
**Node examined**	<12	1	0.111
	≥12	1.072 [0.984-1.167]	
**Gender**	Female	1	0.771
	Male	0.989 [0.915-1.068]	
**Age**	20-59	1	<0.001
	≥60	0.644 [0.594-0.699]	
**Race**	White	1	<0.001
	Black	1.207 [1.084-1.344]	
	Others	1.298 [1.139-1.479]	
**AJCC T Stage**	T1	1	<0.001
	T2	1.291 [1.086-1.534]	
	T3	3.426 [2.988-3.928]	
	T4	5.097 [4.299-6.043]	
**Histological type**	Adenocarcinoma	1	0.494
	Mucous/signet-ring cell	0.928 [0.808-1.067]	
	Others	0.830 [0.446-1.544]	
**Grade**	Grade I/II	1	<0.001
	Grade III/IV	1.910 [1.712-2.131]	

Supplementary Table S3: Univariate and multivariate analyses RCC patients

Characteristic		Univariate analysis		Multivariate analysis	
		HR [95% CI]	P	HR [95% CI]	P
**Gender**	Female	1	0.975		
	Male	0.999 [0.969-1.031]			
**Age**	20-59	1	<0.001	1	<0.001
	≥60	2.435 [2.316-2.561]		2.570 [2.443-2.704]	
**Race**	White	1	<0.001	1	<0.001
	Black	0.955 [0.909-1.002]		1.088 [1.036-1.142]	
	Others	0.697 [0.649-0.749]		0.709 [0.659-0.762]	
**AJCC Stage**	Stage I	1	<0.001	1	<0.001
	Stage II	1.436 [1.373-1.503]		1.480 [1.438-1.496]	
	Stage III	2.335 [2.235-2.440]		2.426 [2.279-2.649]	
**AJCC T Stage**	T1	1	<0.001	1	<0.001
	T2	1.241 [1.156-1.333]		1.226 [1.140-1.318]	
	T3	1.842 [1.734-1.957]		1.969 [1.775-2.185]	
	T4	3.451 [3.222-3.696]		3.394 [3.044-3.783]	
**AJCC N Stage**	N0	1	<0.001	1	<0.001
	N1/2	1.848 [1.792-1.907]		1.264 [1.102-1.450]	
**Histological type**	Adenocarcinoma	1	<0.001	1	<0.001
	Mucous/signet-ring cell	1.156 [1.109-1.206]		1.048 [1.004-1.093]	
	Others	1.917 [1.592-2.309]		1.375 [1.181-1.600]	
**Grade**	Grade I/II	1	<0.001	1	<0.001
	Grade III/IV	1.496 [1.446-1.549]		1.229 [1.186-1.274]	
**Node examined**	<12	1	<0.001	1	0.001
	≥12	0.718 [0.694-0.743]		0.692 [0.668-0.717]	

Supplementary Table S4: Univariate and multivariate analyses for LCC patients

Characteristic		Univariate analysis		Multivariate analysis	
		HR [95% CI]	P	HR [95% CI]	P
**Gender**	Female	1	0.121		
	Male	1.057 [0.986-1.133]			
**Age**	20-59	1	<0.001	1	<0.001
	≥60	2.413 [2.209-2.637]		2.557 [2.338-2.796]	
**Race**	White	1	<0.001	1	<0.001
	Black	1.124 [1.023-1.235]		1.231 [1.120-1.354]	
	Others	0.738 [0.644-0.846]		0.745 [0.650-0.855]	
**AJCC Stage**	Stage I	1	<0.001	1	<0.001
	Stage II	1.644 [1.477-1.829]		1.779 [1.626-1.976]	
	Stage III	2.246 [2.025-2.491]		2.001 [1.730-2.350]	
**AJCC T Stage**	T1	1	<0.001	1	<0.001
	T2	1.430 [1.212-1.686]		1.458 [1.230-1.728]	
	T3	2.113 [1.851-2.412]		2.467 [1.952-3.119]	
	T4	4.041 [3.477-4.697]		4.579 [3.585-5.848]	
**AJCC N Stage**	N0	1	<0.001	1	0.133
	N1/2	1.579 [1.473-1.693]		1.199 [0.946-1.519]	
**Histological type**	Adenocarcinoma	1	<0.001	1	0.354
	Mucous/signet-ring cell	1.292 [1.152-1.448]		1.089 [0.970-1.222]	
	Others	1.119 [0.674-1.858]		1.006 [0.604-1.677]	
**Grade**	Grade I/II	1	<0.001	1	<0.001
	Grade III/IV	1.541 [1.410-1.685]		1.328 [1.213-1.455]	
**Node examined**	<12	1	<0.001	1	<0.001
	≥12	0.813 [0.757-0.872]		0.737 [0.686-0.793]	
